# Lepidium meyenii Walp Exhibits Anti-Inflammatory Activity against ConA-Induced Acute Hepatitis

**DOI:** 10.1155/2018/8982756

**Published:** 2018-12-16

**Authors:** Wei Zheng, Shouwen Du, Mingyao Tian, Wang Xu, Yufei Tian, Tiyuan Li, Yanling Fu, Shipin Wu, Chang Li, Ningyi Jin

**Affiliations:** ^1^Key Laboratory of Jilin Province for Zoonosis Prevention and Control, Institute of Military Veterinary, Academy of Military Medical Science, Changchun 130122, China; ^2^2nd Clinical Medical College of Jinan University, Shenzhen People's Hospital, Shenzhen, China; ^3^College of Veterinary Medicine, Northwest Agriculture and Forestry University, Yangling 712100, China; ^4^Jiangsu Co-Innovation Center for Prevention and Control of Important Animal Infectious Diseases and Zoonoses, Yangzhou, China

## Abstract

Strong inflammation is a prominent pathogenesis of acute hepatitis, which can induce hepatocyte death and lead to liver failure. Lepidium meyenii Walp (Maca) is a traditional herbal medicine mostly used in improving sperm motility and serum hormone levels, etc. However, there are no reports that showed Maca was designed for treating hepatitis so far. Therefore, the protective effects and pharmacological mechanisms of Maca are unknown in hepatitis. In this study, we found that the protective effects of Maca extract ameliorate ConA-induced acute hepatitis (CIH) and underlying mechanisms. We determined that pretreatment with Maca extract significantly suppressed the production of aminotransferases and inflammatory cytokines, including IFN-*γ*, TNF-*α*, IL-1*β*, IL-2, IL-6, IL-12, and IL-17a, and moderated acute liver injury in CIH. Maca recruited more myeloid-derived suppressor cells (MDSCs) to the liver and suppressed infiltration of natural killer T cells (NKT cells) and macrophages in the liver. Furthermore, our data indicated the molecular mechanism of the inhibitory inflammatory effects of Maca, which should suppress the activation of NF-*κ*B, IFN-*γ*/STAT1, and IL-6/STAT3 signalings. Collectively, this present research explores Maca as an effective hepatoprotective medicine to inhibit inflammation and liver injury caused by acute hepatitis.

## 1. Introduction

Hepatitis represents a globally large public health problem in human, which is caused by autoimmune diseases, alcohol consumption, metabolic disorders, viral infections, and fatty liver diseases [1]. Acute hepatitis is characterized by strong inflammation, which induces hepatocyte death and can lead to liver failure; it is therefore a serious threat to human health and life [1, 2]. Concanavalin A- (ConA-) induced hepatitis is an appropriate animal model for drug research and immune-mediated liver injury for human hepatitis [3]. As a potent agent, induction of hepatitis by ConA depends on the release of a broad spectrum of cytokines from immune cells [3]. The inflammatory cytokines, TNF-*α* and IFN-*γ*, play an essential role [4]. Besides, IL-1*β*, IL-2, IL-6, IL-12, and IL-17a are also involved in ConA-induced liver injury [5–7]. A variety of immune cells include T cells, NKT cells, macrophages (yolk sac-derived macrophages (Kupffer cells) and infiltrating macrophages) [8], and neutrophils. In the early stage of ConA injection, CD4^+^ T cells secrete a small amount of cytokines, which activate other proinflammatory immune cells. And then especially, macrophages and NKT cells are the pivotal and direct mediators in ConA-induced liver damage, via cytokine production (TNF-*α*, IFN-*γ*, IL-2, IL-6, and IL-12) and infiltration against hepatocytes [9, 10].

Previous study reports have shown that CIH, which is a type of inflammatory-induced liver injury and is dependent upon NKT cells and macrophages, derived inflammatory cytokines, such as TNF-*α*, IFN-*γ*, and interleukins. These cytokines are regulated by the activation of multiple signaling pathways. NF-*κ*B is a well-known transcription factor that can potently induce proinflammatory mediator secretion during the development of acute liver disease. IFN-*γ*/STAT1 plays a critical role in leukocyte infiltration into the liver and activation of apoptotic signaling pathways in CIH. In IFN-*γ*^−/−^ and STAT1^−/−^ mice, leukocyte infiltrations were significantly suppressed after ConA injection [11]. The blockade of the IL-6/STAT3 signaling pathway ameliorated liver injury [12]. And so, if these relevant pathways and factors can be regulated, that is helpful to reduce hepatitis and liver damage.

Myeloid-derived suppressor cells (MDSCs) represent a common capacity of suppressing immune responses [13]. MDSCs show promising therapeutic targets in the treatment of liver diseases, with the liver being an important site for MDSC accumulation and differentiation under various liver conditions. Interaction of MDSCs and macrophages causes the macrophages to reduce the secretion of IL-12 [14–16]. In acute hepatitis, MDSCs protects against liver injury by suppressing the activation of T cells and macrophages [7]. It is therefore helpful to increase hepatic MDSC numbers for the treatment of patients with acute hepatitis.

At the present time, there are not enough effective drug treatment options for hepatitis. Almost all reports about Maca are used in improving sperm motility and serum hormone levels [17, 18], but the effects of Maca protection against liver damage remains unclear. In this study, we performed experiments to determine the therapeutic effects of Maca on liver inflammation and injury for the first time, and we investigated the cellular and molecular changes in CIH treated by the administration of Maca extract.

## 2. Materials and Methods

### 2.1. Animals and Ethics Statement

All studies were performed according to the National Institutes of Health Guidelines and were also approved by the Institutional Animal Care and Use Committee (IACUC) of the Chinese Academy of Military Medical Sciences (10ZDGG007) and were used in accordance with regulations and guidelines of this committee. All efforts were made to minimize suffering and distress. At the end of the experiment, mice were euthanized by a cervical dislocation method.

Some materials and methods referred to our previously published article [19].

Female BALB/c mice (8–10 weeks old) were obtained from Vital River Lab Animal Technology (Beijing, China. Approval ID: SCXK (Jing) 2016-0011). The mice were housed in an animal facility at a humidity of 40–60% and a temperature of 23 ± 2°C with a 12-hour alternating light and dark cycle. Five mice were housed in a standard polypropylene cage with a stainless steel top grill having facilities such as a drinking water bottle, SPF bedding, and SPF food (Approval IDs: SCXK 2014-0010). The mice were observed once a day. These measures guaranteed any unexpected deaths of mice.

### 2.2. Preparation of Maca Extract

The medicinal Maca (Yunnan Institute of Materia Medica, China) was powdered with a laboratory mechanical grinder. The powdered DH was macerated in anhydrous alcohol and allowed to shake for 4 h and subsequently filtered through three layers of a filter paper. The maceration process was repeated 3 times. The filtered extract was concentrated in a rotary evaporator at 40°C, and then a freeze drier was used to remove ethanol and water. The dry extract was stored at 4°C until use. Maca extract was dissolved in phosphate-buffered saline (PBS) and then was filtered through a 0.22 *μ*m sterile filter (Merck Millipore, IRL). The working concentration of extract is 10 mg/mL.

### 2.3. Analysis of Survival Rate, Transaminase Levels, and Cytokine Levels

Eight to ten-week-old female BALB/c mice were randomly divided into four groups with each group containing 10 mice. The control group mice were injected via the tail vein (i.v.) with PBS. The Maca control group mice were injected by intraperitoneal injection (i.p.) with Maca extract (20 mg/kg of body weight). The ConA group mice were injected i.v. with either a sublethal dose (15 mg/kg) or a lethal dose (30 mg/kg) of ConA, and the working concentration of ConA is 1 mg/mL (Sigma-Aldrich, USA). The Maca-pretreated group mice were injected i.p. with Maca extract (20 mg/kg) 2 hours before injecting i.v. with ConA.

In the survival rate study, mice were monitored every 4 hours after the injection of the lethal dose of ConA and euthanized by a cervical dislocation method at 24 hours postinjection. In the sublethal dose-injected groups, the blood was obtained through orbital plexus bleeding from each group at 8, 16, and 24 hours after ConA administration. It was centrifuged for 5 min (1500×*g*), and the supernatants were saved at −80°C. Serum alanine aminotransferase (ALT) and aspartate aminotransferase (AST) levels were measured by a transaminase kit according to the manufacturer's instructions (Nanjing Jiancheng Bioengineering Institute, Nanjing, China).

As reported has been previously, cytokines reached concentrations at different time points [20]. The blood was obtained through orbital plexus bleeding from each group at 8, 16, and 24 hours after ConA administration. Mice were sacrificed, and the liver tissues were surgically removed at 8, 16, and 24 hours after ConA administration. 400 mg of liver tissue was placed in 800 *μ*L RIPA buffer (high) with PMSF (Beijing Solarbio Science and Technology, Beijing, China) and Phosphorylase inhibitor Cocktail Tablets (Roche LifeScience, Switzerland); then liver tissues were powdered with a tissue mechanical grinder, 60 Hz, 180 seconds. It was centrifuged for 30 min (12,000×*g*), and the supernatants were saved at −80°C. Serum and liver tissue concentrations of TNF-*α*, IFN-*γ*, IL-1*β*, IL-2, IL-6, IL-12, and IL-17a were determined using CBA Flex Sets according to the manufacturer's instructions (BD Biosciences Company, USA).

### 2.4. Western Blot Analysis

Liver tissue supernatants were boiled with 2 × SDS loading buffer for 10 min. The samples were fractionated by electrophoresis on a 12% SDS-PAGE gel and transferred onto PVDF membrane. After blocking with 5% skimmed milk for 4 h, the membrane was incubated with either anti-NF-*κ*B p65, anti-P-NF-*κ*B p65, anti-STAT1, anti-P-STAT1, anti-STAT3, anti-P-STAT3, anti-I*κ*B*α*, anti-P-I*κ*B*α*, or anti-GAPDH (cell signaling technology, USA) and with HRP-conjugated secondary antibodies (DingGuoShengWu, China). Blots were developed using Western HRP substrate. Band intensities were quantified using ImageJ gel analysis software.

### 2.5. TUNEL and Histopathological Study

16 hours after ConA injection, the mice liver injury developed at a significant level [21]. Therefore, we get this time point to examine liver pathology. The mice were euthanized by the cervical dislocation method, and then liver tissues were collected and fixed in 10% buffered neutral formalin for at least 24 hours and embedded in paraffin then prepared. Tissue sections were cut, deparaffinized, and stained with H&E (hematoxylin and eosin) or TUNEL (terminal deoxynucleotidyl transferase- (TdT-) mediated dUTP-biotin nick end labeling) kit (Promega, USA) to observe the level of inflammation and tissue damage by fluorescence microscopy or light microscopy.

### 2.6. Isolation of Liver Mononuclear Cells

Isolation of intrahepatic mononuclear cells (MNCs) was performed as previously described [22]. Briefly, the livers were removed and passed through a 200-gauge stainless steel mesh. The cells were suspended in 42% Percoll and subsequently gently overlaid on 70% Percoll (GE Healthcare Bio-Sciences AB, USA) and centrifuged at 1060×*g* for 25 min at room temperature. Liver MNCs were collected from the interphase of Percoll.

### 2.7. Flow Cytometric Analysis

Macrophages were stained with FITC-anti-CD45 and APC-anti-F4/80, NKT cells were stained with FITC-anti-CD3 and APC-anti-CD49b, and MDSCs were stained with FITC-anti-Gr1 and PE-anti-CD11b monoclonal antibodies for surface antigens according to the standard protocol (Biolegend Inc., USA). The stained cells were analyzed using a flow cytometer (FACScalibur and ACCURI C6; Becton Dickinson, Franklin Lakes, NJ), and the data was analyzed by FlowJo software.

### 2.8. Statistical Analysis

The results were analyzed using GraphPad Prism, version 5.0, by Student's *t*-test or analysis of variance. All data were shown as the mean ± standard error of the mean (SEM). *P* value < 0.05 was considered to be statistically significant.

## 3. Results

### 3.1. Maca Pretreatment Protects Mice against CIH

**Figure 1:**
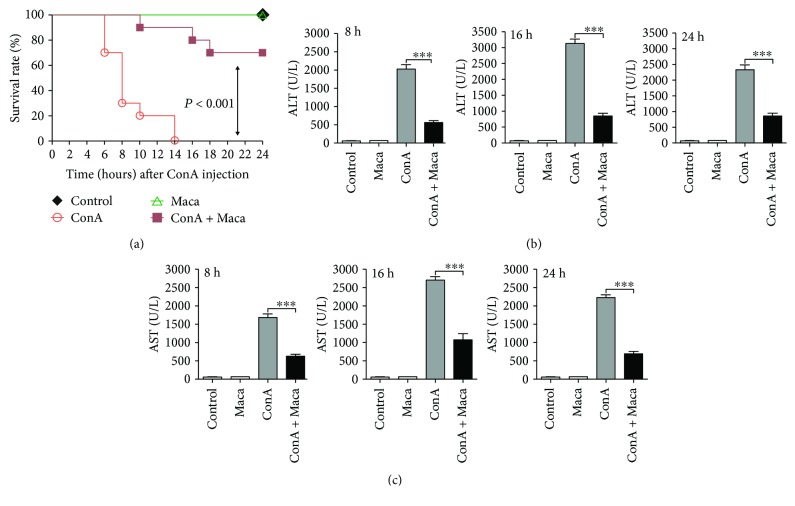
Protection against ConA-induced acute hepatitis. Female BALB/c mice (*n* = 10) were injected i.p. with Maca (20 mg/kg body weight) at 2 hours before the injection of a lethal dose of ConA (30 mg/kg) i.v. (a) The survival rate was monitored at different times after ConA administration. (b, c) Maca suppresses transaminase activity in ConA-induced hepatitis. The sublethal dose of ConA (15 mg/kg of body weight) i.v. at 2 hours after Maca injection i.p. Serum transaminase ALT (b) and AST (c) levels were determined 8, 16, and 24 hours after ConA injection. Data is expressed as the mean ± SD (*n* = 3). ^∗∗∗^*p* < 0.001 vs. the ConA group. ConA, concanavalin A; Maca, Lepidium meyenii Walp; ALT, alanine transaminase; AST, aspartate transaminase.

In order to determine the therapeutic effects of Maca in acute hepatitis, we firstly investigated the effects of Maca on mortality from CIH. Mice were pretreated with Maca and then challenged with a lethal dose of ConA (30 mg/kg). The Maca-pretreated group mice significantly improved the survival rate that were challenged with a lethal dose of ConA, when compared with unpretreated of Maca control group mice (Figure 1(a)). The kinetics of hepatic damage was further determined in mice that were injected i.v. with a sublethal dose of ConA. As an acknowledged marker of hepatic injury [23], blood transaminase was evaluated. As shown in (Figures 1(b) and 1(c)), the levels of serum ALT and AST were revealed significantly higher in the ConA-injected group than in the Maca-pretreated group at 8, 16, and 24 hours after ConA administration. In the meantime, we found that the serums ALT and AST were not different in the Maca-injected group alone compared with the control group, suggesting that Maca did not exhibit drug hepatoxicity. So this increase was obviously reduced by Maca pretreatment, indicating that Maca pretreatment usefully protected against CIH.

**Figure 2:**
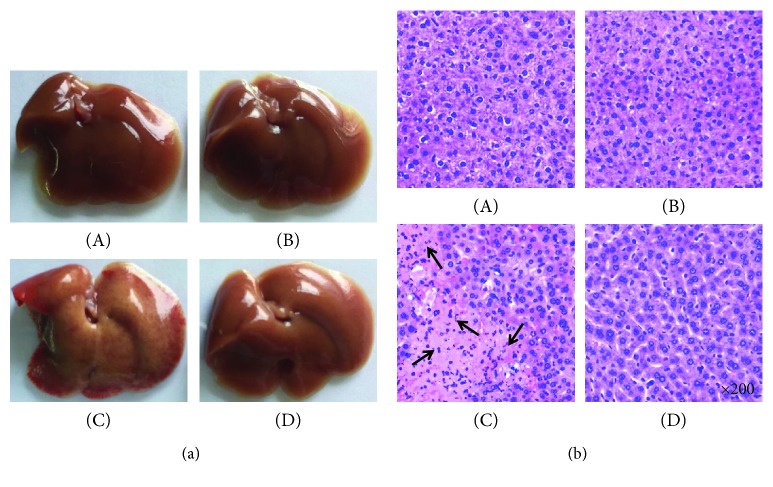
Maca inhibits ConA-induced acute hepatitis. Mice were injected i.p. with DH (20 mg/kg of body weight) at 2 hours before the challenge of ConA (15 mg/kg of body weight). Mice were sacrificed at 16 hours after the ConA injection. The livers were harvested from control ((a), A), Maca ((a), B), ConA ((a), C), and ConA + Maca ((a), D) injection mice, respectively. Liver tissues from the control ((b), A), Maca ((b), B), ConA ((b), C), and ConA + Maca ((b), D) groups were fixed and stained with hematoxylin and eosin (H&E). The arrows indicate massive cell death in the liver section. Original magnification ×200. i.p., intraperitoneal injection; ConA, concanavalin A; Maca, Lepidium meyenii Walp; H&E, hematoxylin and eosin.

**Figure 3:**
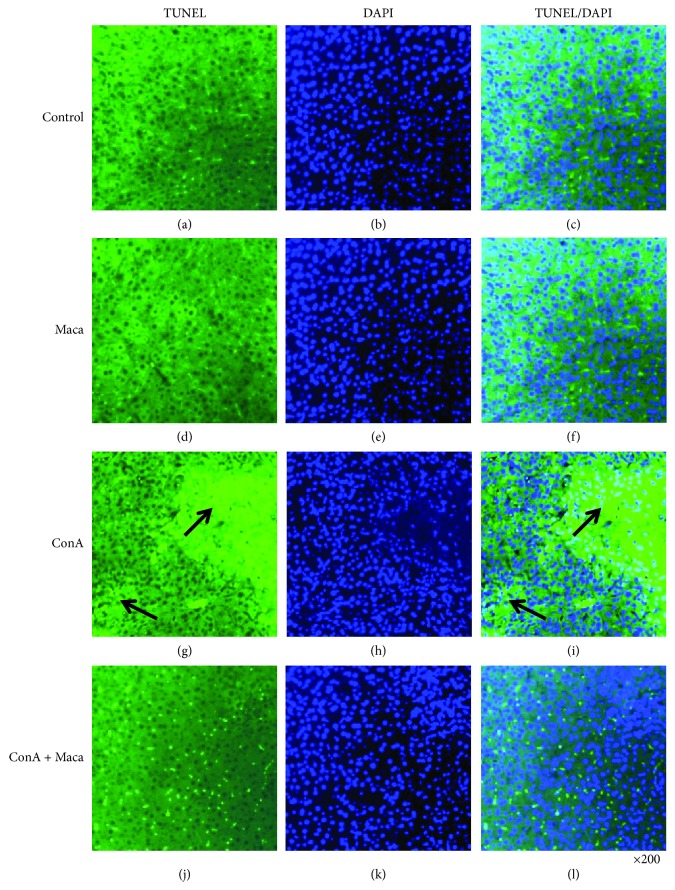
Maca inhibits ConA-induced hepatic apoptosis. Mice were sacrificed at 16 hours after ConA injection. The livers were harvested from the control, Maca, ConA, and ConA + Maca groups. Images showing TUNEL-labeled (green) apoptotic cells counterstained with DAPI (blue) in liver tissue sections. DAPI-stained sections (b, e, h, and k) label the nuclei, while TUNEL-labeling (a, d, g, and j) reveals apoptotic cells. Colocalization of TUNEL/DAPI (c, f, i, and l) in liver sections. The arrows indicate an aggregate of apoptotic cells in the liver section of the ConA injection group. Original magnification ×200. ConA, concanavalin A; Maca, Lepidium meyenii Walp; TUNEL, terminal deoxynucleotidyl transferase- (TdT-) mediated dUTP-biotin nick end labeling; DAPI, 4′,6-diamidino-2-phenylindole.

Anatomical and histological examinations were approved evidence, which were used to evaluate liver injury level (Figure 2(a) shows mice liver photos of different experimental groups, and Figure 2(b) shows H&E staining). The key feature of CIH was inflammatory infiltrates and massive hepatocyte death. More inflammatory infiltrates and massive hepatocyte death were observed in the nonpretreated group. On the contrary, minor inflammatory infiltrates and liver injury were observed in the Maca-pretreated group, and the number of apoptotic cells was obviously reduced as compared with just only the ConA-injected group (Figure 3 shows TUNEL staining). These satisfied findings showed that Maca significantly inhibited liver injury and release of transaminases (ALT and AST) in CIH. In a nutshell, the results showed that Maca effectively protected against liver injury.

### 3.2. Production of Inflammatory Cytokines Is Inhibited by Maca in CIH

**Figure 4:**
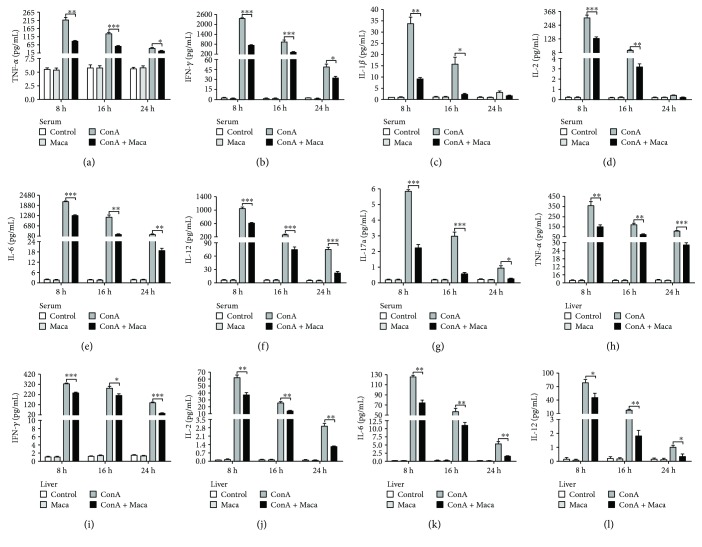
Maca inhibits expression of proinflammatory cytokines in ConA-induced hepatitis. Blood and liver tissue samples were collected from the control, Maca-treated, ConA, and ConA + Maca groups at 8, 16, and 24 hours after ConA injection. (a–g) Serum concentration of TNF-*α*, IFN-*γ*, IL-1*β*, IL-2, IL-6, IL-12, and IL-17a and (h–l) liver tissue concentration of TNF-*α*, IFN-*γ*, IL-2, IL-6, and IL-12 were determined using CBA Flex Sets. The similar results are representative of three experiments. Data is expressed as the mean ± SD (*n* = 3). ^∗^*p* < 0.05, ^∗∗^*p* < 0.01, and ^∗∗∗^*p* < 0.001 vs. the ConA group. ConA, concanavalin A; Maca, Lepidium meyenii Walp; IFN-*γ*, interferon-*γ*; TNF-*α*, tumor necrosis factor alpha; IL-1*β*, interleukin-1*β*; IL-2, interleukin-2; IL-6, interleukin-6; IL-12, interleukin-12; IL-17a, interleukin-17a.

Previously, studies showed that following ConA administration, immune cells were activated. Accordingly, the releasing of various inflammatory cytokines played critical roles to aggravate in acute hepatic injury, including IFN-*γ*, TNF-*α*, IL-2, IL-6, IL-12, and other cytokines [24]. To explore the underlying mechanisms of Maca protection against liver injury, the effects of Maca in various inflammatory cytokine productions in CIH were studied. We examined serum and liver tissue level of TNF-*α*, IFN-*γ*, IL-1*β*, IL-2, IL-6, IL-12, and IL-17a by CBA Flex Set. The results were satisfactory. Not only in serum but also in liver tissue, Maca pretreatment significantly inhibited the level of inflammatory cytokines, including TNF-*α*, IFN-*γ*, IL-1*β*, IL-2, IL-6, IL-12, and IL-17a in serum and TNF-*α*, IFN-*γ*, IL-2, IL-6, and IL-12 in liver tissue in ConA-challenged mice (Figures 4(a)–4(l)). Thus, the above results indicated that Maca might commendably suppress the expression of multiple inflammatory cytokines in CIH.

### 3.3. More MDSCs Are Recruited by Maca to the Liver against CIH

**Figure 5:**
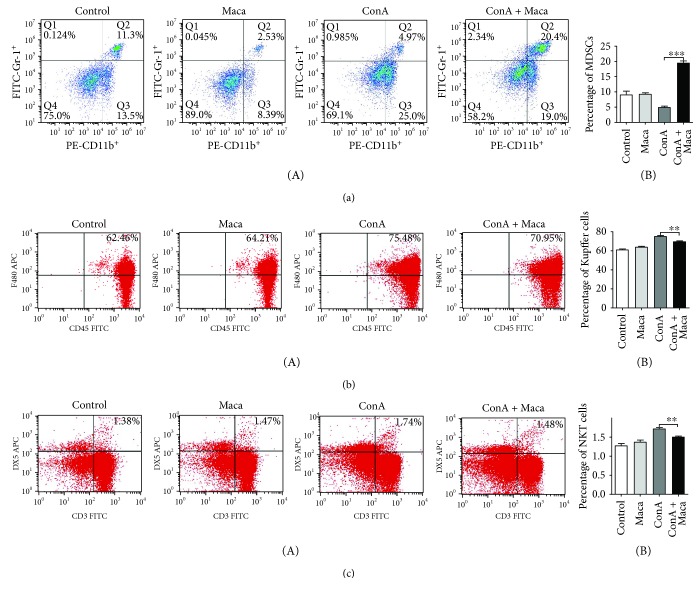
Maca recruits more MDSCs to the liver and inhibits the infiltration of Kupffer cells and NKT cells in ConA-induced hepatitis. Hepatitis was induced by i.v. injection of ConA (15 mg/kg). Liver mononuclear cells were isolated from mice at 16 hours following ConA injection. (a) PE-CD11b^+^ FITC-Gr1^+^ MDSCs, (b) FITC-CD45^+^ APC-F4/80^+^ Kupffer cells, and (c) FITC-CD3^+^ APC-DX5^+^ NKT cells were stained and analyzed by a flow cytometer. Numbers in quadrants indicate the percentage of cells in each (a-B), (b-B), and (c-B). Proportions of (a-B) PE-CD11b^+^ FITC-Gr1^+^ MDSCs, (b-B) FITC-CD45^+^ APC-F4/80^+^ Kupffer cells, and (c-B) FITC-CD3^+^ APC-DX5^+^ NKT cells among total liver mononuclear cell population. The similar results are representative of three experiments. Data is expressed as the mean ± SD (*n* = 3). ^∗∗^*p* < 0.01 and ^∗∗∗^*p* < 0.001 vs. the ConA group. ConA, concanavalin A; Maca, Lepidium meyenii Walp; MDSCs, myeloid-derived suppressor cells; NKT cells, natural killer T cells.

CIH is a type of inflammatory-induced liver injury and is dependent upon T cells, NKT cells, and macrophage-derived inflammatory cytokines [3, 25]. MDSCs present a common capacity of suppressing immune cell responses, including inhibition of infiltrating macrophages and T cell activation, and so on, which can maintain the immune homeostasis in the liver [13]. If Maca can effectively prevent the infiltration and activation of NKT cells and macrophages in time, it will be possible to reduce liver damage in CIH. We examined whether Maca affects the recruitment and activation of MDSCs in the liver under inflammatory conditions. Indeed, we found that the percentage of CD11b^+^ Gr-1^+^ MDSCs in the Maca-pretreated mouse liver was remarkably higher than that in the ConA-injected group (Figure 5(a)). Meanwhile, we also found that the percentage of CD45^+^ F4/80^+^ macrophages and CD3^+^ DX5^+^ NKT cells in the ConA group was much higher than that in the Maca-pretreated group (Figures 5(b) and 5(c)). Our results seem to be consistent with the previously reported findings. All of these indicated that Maca might recruit more MDSCs to the liver and inhibit macrophages and NKT cell infiltration and inflammatory responses to reduce liver injury.

### 3.4. Maca Pretreatment Inhibited Activation of NF-*κ*B, STAT1, and STAT3 in CIH

**Figure 6:**
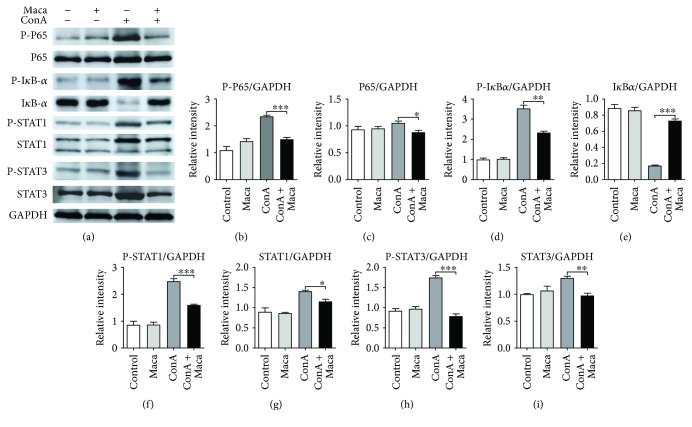
Inhibitory effects of Maca on liver damage and apoptosis-related protein expression in ConA-induced acute hepatitis. Liver tissues were collected from mice 16 hours after ConA challenge and analyzed by Western blot. (a) Effects of Maca on P-P65, P65, P-I*κ*B-*α*, I*κ*B-*α*, P-STAT1, STAT1, P-STAT3, and STAT3 expression were confirmed by Western blot. (b–i) Quantification of relative protein expression was performed by densitometric analysis. GAPDH was used as internal control. The similar results are representative of three experiments. Data is expressed as the mean ± SD (*n* = 3). ^∗^*p* < 0.05, ^∗∗^*p* < 0.01, and ^∗∗∗^*p* < 0.001 vs. the ConA group. ConA, concanavalin A; Maca, Lepidium meyenii Walp; P-P65, phosphorylated P65; P-I*κ*B-*α*, phosphorylated nuclear factor of kappa light polypeptide gene enhancer in B-cells inhibitor alpha; I*κ*B-*α*, nuclear factor of kappa light polypeptide gene enhancer in B-cells inhibitor alpha; P-STAT1, phosphorylated signal transducer and activator of transcription 1; STAT1, signal transducer and activator of transcription 1; P-STAT3, phosphorylated signal transducer and activator of transcription 3; STAT3, signal transducer and activator of transcription 3.

NF-*κ*B is a well-known transcription factor that plays a critical role in the activation of several inflammatory pathways. The effect of Maca on anti-inflammatory was evaluated to detect the NF-*κ*B pathway activation. As presented in Figures 6(a)–6(e), Maca pretreatment obviously reduced P65 phosphorylation and blocked I*κ*B*α* phosphorylation and degradation, compared with that in the ConA administration group. These results suggested that part of the reason for the inhibition of inflammatory response by Maca might be the block of NF-*κ*B signaling pathway activation.

In the meantime, IFN-*γ*/STAT1 acts as a proinflammatory signal pathway, which is crucial for CIH [11]. The synthesis and activation of STAT1 are increased in the liver of ConA-induced hepatitis mice, and STAT1-deficient mice attenuate ConA-induced liver damage [11, 26]. Therefore, we envisaged whether Maca affects the IFN-*γ*/STAT1 signaling pathway in ConA-induced liver injury. Indeed, in our studies, we found that Maca effectively reduced synthesis and phosphorylation of STAT1 (Figures 6(a), 6(f), and 6(g)), indicating that Maca decreased synthesis and activation of STAT1 was also a factor of the inhibition of hepatocyte necrosis induced by ConA challenge.

Excessive IL-6 was described to be hepatodestructive when injected or increased after ConA injection [27]. In mouse in vivo, inflammatory responses were triggered through the IL-6/STAT3 signaling pathway, and protein expression of total STAT3 and phosphorylation of STAT3 were enhanced after ConA administration [28]. Our previous results showed Maca pretreatment significantly inhibited IL-6 levels of serum and liver tissue compared with the ConA-injected group. Thus, Maca pretreatment might have achieved the effect of inhibiting liver injury by reducing the synthesis and phosphorylation of STAT3. The results were in line with our expectation; we found that Maca markedly decreased STAT3 synthesis and ConA-induced STAT3 phosphorylation (Figures 6(a), 6(h), and 6(i)), indicating that inhibition of inflammatory responses by Maca might be partially responsible for suppressing the synthesis and phosphorylation of the IL-6/STAT3 signaling pathway.

## 4. Discussion

Following an increasing number of studies, traditional herbal medicines exactly play an important role in the types of disease therapies: tumor, hepatitis, autoimmune disease, viral disease, and so on. Herbs touted as potential therapeutic protection against liver disease have been reported [29]. Maca, one of the popular herbs, has been used in improving sperm motility and serum hormone levels around the world for hundreds of years [17, 18], but there is no research to report that Maca would prevent hepatitis, not to mention the underlying molecular mechanism.

ConA-induced acute hepatitis is a classic mouse model of immune-meditated liver injury, which exhibits an acute elevation of blood aminotransferase, secretion of proinflammatory cytokines, and infiltration of inflammatory cells [3, 30]. In the base of our study, the results demonstrate the therapeutic effect of Maca in ConA-induced acute hepatitis and provide novel evidences regarding its new territory of pharmacological properties. When liver cells are injured, transaminases are released from the liver cells into the blood. So ALT and AST were known as one metric to assess the severity of liver injury. When ConA injection is reduced to a sublethal dose, our results showed that the ALT/AST levels were suppressed and decreased liver damage was decreased in the Maca-pretreated group. After ConA injection, anatomical liver photos, H&E, and TUNEL significantly proved that liver cell necrosis, inflammatory cell infiltration, and liver cell apoptosis were remarkably reduced in Maca9-pretreated mice. These findings showed that Maca pretreatment alleviated pathological changes, suggesting potential for the use of Maca in clinical application to prevent liver injury though inhibition of inflammation damage. Furthermore, we found that some of the apoptosis signals (bright-green nuclei) were still present in the ConA + Maca group of the TUNEL image. We consider that the apoptosis cells were not only liver cells but also multiple proinflammatory immune cellular. If Maca could interact directly with certain proinflammatory cells and cause proinflammatory cell apoptosis, then inflammation-mediated liver damage could be inhibited. It may be another way to reduce the inflammatory response. Surely, these ideas also require a lot of experimental data to prove in future research.

Autoimmune liver damage is associated with excessive activation of the immune responses [31]. Previous studies have reported that the pathogenesis of CIH is mainly due to the release of proinflammatory cytokines, secreted from activated infiltrating macrophages and T cells, including TNF-*α*, IFN-*γ*, IL-1*β*, IL-2, IL-6, IL-12, IL-17a, and other inflammatory cytokines. TNF-*α* is a major player in inflammation-mediated hepatocyte death [32]. Mice injected with a TNF-*α* inhibitor or anti-TNF-*α* and mice deficient in TNF-*α* receptor are effective against CIH. Destruction of IFN-*γ* genes abolished elevated transaminase activities and necrosis in CIH [30]. Our results showed that not only in the serum and but also in the liver tissues Maca might significantly reduce the production of multiple proinflammatory cytokines, including IFN-*γ*, TNF-*α*, IL-1*β*, IL-2, IL-6, IL-12, and IL-17a.

Macrophage-secreted TNF-*α* and NKT-secreted IFN-*γ* play dominant roles in CIH [33–35]. Once macrophages are depleted and the secretion of TNF-*α* decreased significantly, liver damage is also completely suppressed [36, 37]. NKT cells are the central mediator in ConA-induced liver injury, via IFN-*γ* production and direct cytotoxicity against hepatocytes [9, 10]. Therefore, the activation and infiltration of macrophages and NKT cells are inhibited, which will relieve liver injury. In this study, we found Maca effectively suppressed CD45^+^ F4/80^+^ macrophages and CD3^+^ DX5^+^ NKT cell activation and infiltration in the liver. It has been reported that CD11b^+^ Gr-1^+^ MDSCs suppress the activation and function of macrophages and T cells [14, 38, 39]. As a drug, FTY720 was used to treat autoimmune hepatitis, inhibits Th1 cells to produce IFN-*γ*, activates Foxp3^+^ Tregs by recruiting MDSCs, and reduces liver damage in CIH [40]. MDSCs downregulate the production of TNF-*α* and IL-12 [16, 41]. So we thought about whether Maca acted directly on macrophages and NKT cells or indirectly regulated macrophages and NKT cells by recruiting and activating MDSCs. Our results showed that Maca should recruit more MDSCs to the liver firstly and then inhibited the activation and infiltration of macrophages and NKT cells, leading to reduce the production of large amounts of proinflammatory cytokines.

Based on our above findings, we further explored the molecular mechanism of Maca against ConA-induced inflammatory responses. First, we focused on NF-*κ*B, a key transcription factor involved in inflammatory responses and various autoimmune disease. It can strongly induce proinflammatory mediators in the development of CIH [42]. Our results indicated that Maca pretreatment effectively inhibited P65 and I*κ*B*α* phosphorylation, blocking I*κ*B*α* degradation, suggesting that Maca might block NF-*κ*B activation and subsequently attenuate the transcription of target proinflammatory cytokine genes, thus protecting mice against CIH. IFN-*γ*/STAT1 acts as a proinflammatory signal pathway, which is crucial for CIH [11]. As a major cytokine responsible for the activation of STAT1, IFN-*γ* induces phosphorylation of STAT1, which causes the expression of proapoptotic genes [30]. Blocking IFN-*γ* may prevent ConA-induced damage [43]. In this study, we found that synthesis and phosphorylation of STAT1 were attenuated by Maca pretreatment in the liver. This echoed our above results, in which Maca decreased the production of IFN-*γ* and reduced liver damage. In mouse in vivo, inflammatory responses were triggered through the IL-6/STAT3 signaling pathway, and protein expression of total STAT3 and phosphorylation of STAT3 were enhanced after ConA administration [28]. Excessive accumulation of IL-6 activated STAT3 in liver, and phosphorylation of STAT3 translocated to the nucleus where it regulated multiple inflammatory cytokine gene expressions. IL-6-mediated activation of STAT3 has been implicated in several acute inflammatory diseases [44]. Blockade of IL-6/STAT3 signaling ameliorated liver injury [12]. Consistent with these results, our results showed that pretreatment with Maca significantly reduced the expression of STAT3 and inhibited STAT3 phosphorylation in the liver, indicating Maca had a potential role to inhibit the IL-6/STAT3 signaling pathway in CIH.

In summary, we used a ConA-induced liver injury model to display the effect of Maca on acute hepatitis. Our data showed that Maca played a critical role in protecting the liver by inhibiting inflammatory and immune responses via recruiting more MDSCs in CIH. Furthermore, the underlying mechanisms should be closely associated with inhibition of NF-*κ*B activation and IFN-*γ*/STAT1 and IL-6/STAT3 signaling pathways. Taken together, our beneficial results provide the new pharmacodynamic evidence for the application of Maca in preventing the liver from inflammatory damage.

## Data Availability

The data used to support the findings of this study are available from the corresponding author upon request.

## References

[1] Heneghan M. A., McFarlane I. (2002). Current and novel immunosuppressive therapy for autoimmune hepatitis. *Hepatology*.

[2] Stan S. D., Singh S. V., Brand R. E. (2010). Chemoprevention strategies for pancreatic cancer. *Nature Reviews Gastroenterology & Hepatology*.

[3] Tiegs G., Hentschel J., Wendel A. (1992). A T cell-dependent experimental liver injury in mice inducible by concanavalin A. *The Journal of Clinical Investigation*.

[4] Wolf D., Hallmann R., Sass G. (2001). TNF-*α*-induced expression of adhesion molecules in the liver is under the control of TNFR1—relevance for concanavalin A-induced hepatitis. *Journal of Immunology*.

[5] Takahashi K., Murakami M., Kikuchi H., Oshima Y., Kubohara Y. (2011). Derivatives of *Dictyostelium* differentiation-inducing factors promote mitogen-activated IL-2 production via AP-1 in Jurkat cells. *Life Sciences*.

[6] Nicoletti F. (2000). Murine concanavalin A-induced hepatitis is prevented by interleukin 12 (IL-12) antibody and exacerbated by exogenous IL-12 through an interferon-*γ*-dependent mechanism. *Hepatology*.

[7] Diao W., Jin F., Wang B. (2014). The protective role of myeloid-derived suppressor cells in concanavalin A-induced hepatic injury. *Protein & Cell*.

[8] Zigmond E., Samia-Grinberg S., Pasmanik-Chor M. (2014). Infiltrating monocyte-derived macrophages and resident kupffer cells display different ontogeny and functions in acute liver injury. *Journal of Immunology*.

[9] Kaneko Y., Harada M., Kawano T. (2000). Augmentation of V*α*14 NKT cell-mediated cytotoxicity by interleukin 4 in an autocrine mechanism resulting in the development of concanavalin A-induced hepatitis. *The Journal of Experimental Medicine*.

[10] Takeda K., Hayakawa Y., van Kaer L., Matsuda H., Yagita H., Okumura K. (2000). Critical contribution of liver natural killer T cells to a murine model of hepatitis. *Proceedings of the National Academy of Sciences of the United States of America*.

[11] Jaruga B., Hong F., Kim W. H., Gao B. (2004). IFN-*γ*/STAT1 acts as a proinflammatory signal in T cell-mediated hepatitis via induction of multiple chemokines and adhesion molecules: a critical role of IRF-1. *American Journal of Physiology. Gastrointestinal and Liver Physiology*.

[12] Yamaguchi K., Itoh Y., Yokomizo C. (2011). Blockade of IL-6 signaling exacerbates liver injury and suppresses antiapoptotic gene expression in methionine choline-deficient diet-fed db/db mice. *Laboratory Investigation*.

[13] Gabrilovich D. (2004). Mechanisms and functional significance of tumour-induced dendritic-cell defects. *Nature Reviews. Immunology*.

[14] Ostrand-Rosenberg S., Sinha P. (2009). Myeloid-derived suppressor cells: linking inflammation and cancer. *Journal of Immunology*.

[15] Gabrilovich D. I., Nagaraj S. (2009). Myeloid-derived suppressor cells as regulators of the immune system. *Nature Reviews. Immunology*.

[16] Sinha P., Clements V. K., Bunt S. K., Albelda S. M., Ostrand-Rosenberg S. (2007). Cross-talk between myeloid-derived suppressor cells and macrophages subverts tumor immunity toward a type 2 response. *Journal of Immunology*.

[17] Uchiyama F., Jikyo T., Takeda R., Ogata M. (2014). *Lepidium meyenii* (Maca) enhances the serum levels of luteinising hormone in female rats. *Journal of Ethnopharmacology*.

[18] Melnikovova I., Fait T., Kolarova M., Fernandez E. C., Milella L. (2015). Effect of *Lepidium meyenii* Walp. on semen parameters and serum hormone levels in healthy adult men: a double-blind, randomized, placebo-controlled pilot study. *Evidence-Based Complementary and Alternative Medicine*.

[19] Zheng W., Wang Q., Lu X. (2016). Protective effects of *Dracocephalum heterophyllum* in ConA-induced acute hepatitis. *Mediators of Inflammation*.

[20] Kusters S., Gantner F., Kunstle G., Tiegs G. (1996). Interferon gamma plays a critical role in T cell-dependent liver injury in mice initiated by concanavalin A. *Gastroenterology*.

[21] Shen M., Lu J., Cheng P. (2014). Ethyl pyruvate pretreatment attenuates concanavalin A-induced autoimmune hepatitis in mice. *PLoS One*.

[22] Wang J., Sun R., Wei H., Dong Z., Gao B., Tian Z. (2006). Poly I:C prevents T cell-mediated hepatitis via an NK-dependent mechanism. *Journal of Hepatology*.

[23] Kim S. J., Lee S. M. (2013). NLRP3 inflammasome activation in D-galactosamine and lipopolysaccharide-induced acute liver failure: role of heme oxygenase-1. *Free Radical Biology & Medicine*.

[24] Kusters J. G., Gerrits M. M., van Strijp J., Vandenbroucke-Grauls C. M. (1997). Coccoid forms of Helicobacter pylori are the morphologic manifestation of cell death. *Infection and Immunity*.

[25] Wang H. X., Liu M., Weng S. Y. (2012). Immune mechanisms of Concanavalin A model of autoimmune hepatitis. *World Journal of Gastroenterology*.

[26] Siebler J., Wirtz S., Klein S. (2003). A key pathogenic role for the STAT1/T-bet signaling pathway in T-cell-mediated liver inflammation. *Hepatology*.

[27] Tagawa Y., Matthys P., Heremans H. (2000). Bimodal role of endogenous interleukin-6 in concanavalin A-induced hepatitis in mice. *Journal of Leukocyte Biology*.

[28] Akla N., Pratt J., Annabi B. (2012). Concanavalin-A triggers inflammatory response through JAK/STAT3 signalling and modulates MT1-MMP regulation of COX-2 in mesenchymal stromal cells. *Experimental Cell Research*.

[29] Ding R. B., Tian K., huang L. L. (2012). Herbal medicines for the prevention of alcoholic liver disease: a review. *Journal of Ethnopharmacology*.

[30] Hong F., Jaruga B., Kim W. H. (2002). Opposing roles of STAT1 and STAT3 in T cell-mediated hepatitis: regulation by SOCS. *The Journal of Clinical Investigation*.

[31] Rolando N. (2000). The systemic inflammatory response syndrome in acute liver failure. *Hepatology*.

[32] Gantner F., Leist M., Lohse A. W., Germann P. G., Tiegs G. (1995). Concanavalin A-induced T-cell-mediated hepatic injury in mice: the role of tumor necrosis factor. *Hepatology*.

[33] Antoniades C. G., Berry P. A., Wendon J. A., Vergani D. (2008). The importance of immune dysfunction in determining outcome in acute liver failure. *Journal of Hepatology*.

[34] Mizuhara H., O'Neill E., Seki N. (1994). T cell activation-associated hepatic injury: mediation by tumor necrosis factors and protection by interleukin 6. *The Journal of Experimental Medicine*.

[35] Chen S., Akbar S. M. F., Abe M., Hiasa Y., Onji M. (2011). Immunosuppressive functions of hepatic myeloid-derived suppressor cells of normal mice and in a murine model of chronic hepatitis B virus. *Clinical and Experimental Immunology*.

[36] Bilzer M., Roggel F., Gerbes A. L. (2006). Role of Kupffer cells in host defense and liver disease. *Liver International*.

[37] Yamano T., DeCicco L. A., Rikans L. E. (2000). Attenuation of cadmium-induced liver injury in senescent male fischer 344 rats: role of Kupffer cells and inflammatory cytokines. *Toxicology and Applied Pharmacology*.

[38] Ray P., Arora M., Poe S. L., Ray A. (2011). Lung myeloid-derived suppressor cells and regulation of inflammation. *Immunologic Research*.

[39] Bunt S. K., Yang L., Sinha P., Clements V. K., Leips J., Ostrand-Rosenberg S. (2007). Reduced inflammation in the tumor microenvironment delays the accumulation of myeloid-derived suppressor cells and limits tumor progression. *Cancer Research*.

[40] Liu G., Bi Y., Wang R. (2014). Targeting S1P1 receptor protects against murine immunological hepatic injury through myeloid-derived suppressor cells. *Journal of Immunology*.

[41] Kelly M. G., Alvero A. B., Chen R. (2006). TLR-4 signaling promotes tumor growth and paclitaxel chemoresistance in ovarian cancer. *Cancer Research*.

[42] Li Q., Verma I. M. (2002). NF-*κ*B regulation in the immune system. *Nature Reviews. Immunology*.

[43] Tagawa Y., Sekikawa K., Iwakura Y. (1997). Suppression of concanavalin A-induced hepatitis in IFN-*γ*-/- mice, but not in TNF-*α*-/- mice: role for IFN-*γ* in activating apoptosis of hepatocytes. *Journal of Immunology*.

[44] Fielding C. A., McLoughlin R. M., McLeod L. (2008). IL-6 regulates neutrophil trafficking during acute inflammation via STAT3. *Journal of Immunology*.

